# Oxidative Stress, Epigenetics, Environment, and Epidemiology of Diabetic Retinopathy

**DOI:** 10.1155/2017/6419357

**Published:** 2017-02-22

**Authors:** Goran Petrovski, Kai Kaarniranta, Daniel Petrovič

**Affiliations:** ^1^Stem Cells and Eye Research Laboratory, Department of Ophthalmology, Faculty of Medicine, Albert Szent-Györgyi Clinical Center, University of Szeged, Szeged, Hungary; ^2^Centre of Eye Research, Department of Ophthalmology, Oslo University Hospital and University of Oslo, Oslo, Norway; ^3^Department of Ophthalmology, University of Eastern Finland and Kuopio University Hospital, Kuopio, Finland; ^4^Faculty of Medicine, University of Ljubljana, Ljubljana, Slovenia

Several environmental (external) and genetic (internal) factors are involved in the development of diabetic retinopathy (DR). Proper screening and early detection of such factors shall improve the prevention and treatment of this blinding disease. Lately, oxidative stress and factors influencing it have been recognised as important determinants of DR appearance and fulmination. Recently, beside genetical factors, epigenetic mechanisms (i.e., global acetylation of retinal histone H3) have been demonstrated to play an important role in the development and progression of DR to more severe and vision threatening stages, proliferative diabetic retinopathy and diabetic macular edema. Other internal factors affecting DR determined by exome sequencing may prove very helpful in the evaluation of diabetic retinopathy, while screening for external factors may supplement prevention or early diagnosis of the disease. Furthermore, new treatment modalities in microvascular disorders such as DR are emerging that may affect oxidative stress.

The articles contained in the present issue include both reviews and basic scientific studies focused on characterizing DR.

In the work by D. J. Eszes et al. entitled “Diabetic Retinopathy Screening Using Telemedicine Tools: Pilot Study in Hungary,” the authors showed that telemedicine could be a strong method, supporting eye care professionals and allowing for faster and more comfortable DR screening. Moreover, participants found digital retinal screening to be reliable and comfortable. Thirty percent of the patients had never participated in any ophthalmological screening. From all patients examined, 25.7% had DR based upon a standard fundus camera examination and a UK-based DR grading protocol (SpectraTM software). Additionally, there was a statistically significant relationship between economic activity, education and marital status, and future interest of participation. The study represents part of a larger Diabetic Retinopathy Initiative supported by EURETINA in the pilot city, Szeged, Hungary.

In the work by S. Vujosevic and E. Midena entitled “Diabetic Retinopathy in Italy: Epidemiology Data and Telemedicine Screening Programs,” the authors reviewed the available epidemiological data on DR and telematic screening realities in Italy. In Italy, the number of people living with diabetes is about 3.5 million (5.5% of the population), with an increase by about 60% in the last 20 years, and 1 person out of 3 is older than 65 years. The Italian Health Service system estimates that 10 billion euros are spent annually on caring for patients with diabetes, a figure that increases yearly. At the present time, the use of telemedicine for the screening of DR in Italy is confined to geographically limited locations. They concluded, however, that telemedicine might be helpful for establishing a national screening program in Italy.

In the work by A. Horwitz et al. entitled “Danish Nationwide Data Reveal a Link between Diabetes Mellitus, Diabetic Retinopathy, and Glaucoma,” the authors showed that the use of diabetic drugs was strongly associated with the use of antiglaucomatous drugs, while the presence of DR and/or joint complications with DR and nephropathy increases the risk of glaucoma. Moreover, concomitant antihypertensive medication was overall associated with an increased risk of glaucoma. However, the combination of *β*-blocker and renin-angiotensin system inhibitors appeared to have a significantly lower hazard ratio for glaucoma onset in subjects with diabetes. They concluded that a strong association between DM and treatment for glaucoma was shown in the entire Danish population.

In the work by S. Vavuli et al. entitled “Elevated Levels of Plasma IgA Autoantibodies against Oxidized LDL Found in Proliferative Diabetic Retinopathy but Not in Nonproliferative Retinopathy,” the authors showed that IgA autoantibodies were increased in proliferative DR, especially in Type 2 diabetes. The high levels of IgA reflect the inflammatory process and enlighten the role of oxLDL and its autoantibodies in proliferative DR.

In the work by S. S. Pukl et al. entitled “Visual Acuity, Retinal Sensitivity, and Macular Thickness Changes in Diabetic Patients without Diabetic Retinopathy after Cataract Surgery,” the authors showed that underlying diabetes has no effect upon the surgical outcome of cataract surgery in patients with diabetes and having no DR. Best corrected visual acuity (BCVA) measured by ETDRS letters improved significantly and similarly in subjects with diabetes in comparison to subjects without diabetes. However, slight thickening of wider macula and corresponding decrease in retinal sensitivity observed in patients with diabetes 6 months postoperatively might influence visual function on the long term.

In the work by P. Romero-Aroca et al. entitled “Diabetic Macular Edema Pathophysiology: Vasogenic versus Inflammatory,” the authors addressed the pathogenesis of diabetic macular edema. In the paper they reviewed the data currently available, focusing on vascular endothelial growth factor (VEGF), angiogenesis, and inflammation.

